# Where are the paediatric patients with testicular torsion during the COVID-19 pandemic?

**DOI:** 10.1007/s11845-021-02803-z

**Published:** 2021-10-18

**Authors:** Sarah Marie Norton, Shane Considine, Catherine Dowling, Frank D’Arcy

**Affiliations:** Department of Urology, Galway University Hospital, Newcastle Road, Galway, Ireland

**Keywords:** Coronavirus, COVID, Emergency, Paediatric, Torsion

## Abstract

**Introduction:**

The Irish people were put on lockdown in mid-March 2020 due to concern of the spread of coronavirus. With these societal changes came a notable reduction in emergency department attendance. Our aim was to analyse emergency urological procedures performed during the COVID-19 era versus the previous year.

**Methods:**

A retrospective review of theatre logbooks was undertaken comparing numbers of emergency urological procedures performed between 1 March 2020 and 31 May 2020 (i.e. the COVID-19 era) with the corresponding 3-month period in 2019.

**Results:**

A total of 173 cases were analysed between the two time periods. Similar overall numbers of cases were performed in 2019 (*n* = 90) and 2020 (*n* = 83). In particular, similar patient case numbers are also noted in both scrotal explorations (13 vs 9) and ureteric stone surgeries (69 vs 70). However, orchidectomies for testicular cancers were reduced by 63% (3/8). On further analysis of the scrotal exploration group, only 3 were performed in the period after lockdown regulations were instated.

**Conclusion:**

Whilst patients with ureteric colic continue to present, those with acute testis pain requiring exploration attended less frequently, raising the possibility of undiagnosed testicular torsion in the community.

## Introduction

The Irish government acted decisively in mid-March 2020 to put the country in lockdown due to concern regarding COVID-19 spreading throughout communities. Overnight, drastic changes in how we live as a society occurred. Schools and universities closed, non-essential workplaces closed and across the country, the people of Ireland were advised to stay at home and socially distance from other members of their communities.

With these social changes, adaptations to how hospital services routinely run were also undertaken in order to limit the spread of the coronavirus. Specifically, in Galway University Hospital, these changes included two separate emergency department pathways, one for COVID-19 suspected patients and the other for non-COVID-19 emergency presentations; the use of virtual telephone consultations for any patients that did not need to be directly reviewed in the outpatient setting and the cancellation of many elective lists at the start of the pandemic with new pathways put in place for urgent cancer surgeries.

Our department was reconfigured so that patients being referred with a potential urological issue were seen directly by the urology service to ensure the appropriate care of the patient. Worryingly there was an overall reduction in emergency department attendances in the initial lockdown phase. This is in line with previously reported reductions in emergency department attendances of between 73 and 88% in paediatric patients [1] internationally, with a reduction of 50% noted in a paediatric hospital in Ireland [2].

Our aim was to compare the number of acute urological emergency surgeries and orchidectomies being performed during COVID-19 lockdown period, with the corresponding time period in the previous year. Our concern was that patients with an acute urological emergency may be deferring attendance to our emergency department.

## Methods

A retrospective review of urology theatre logbooks in Galway University Hospital was undertaken. We compared numbers of procedures performed between 1 March 2020 and 31 May 2020 (i.e. the COVID-19 era) with the corresponding 3-month period in 2019. These time periods were chosen to mitigate seasonal variations in presentations. Emergency scrotal explorations for testicular pain, orchidectomies performed for testicular cancer and acute ureteric colic requiring ureteroscopy, with or without LASER and JJ stent insertion, were all compared. Elective surgical interventions were not considered in this study.

## Results

A total of 173 cases were analysed between the two time periods (Table 1). One patient in 2020 was excluded from scrotal exploration data as it was an older patient who required an orchidectomy due to severe epididymo-orchitis not responding to antibiotic treatment. No other cases were excluded.

**Table 1:** The comparison of cases in 2019 and 2020

	**2019**	**2020**
Scrotal explorations	13	9*
Ureteric stone surgeries	69	70
Orchidectomies	8	3
**Total**	**90**	**83**

*6 pre-lockdown, 3 post-lockdown.

Similar overall numbers of cases were performed in 2019 (*n* = 90) and 2020 (*n* = 83). In particular, similar patient case numbers are also noted in both scrotal explorations (13 vs 9) and ureteric stone surgeries (69 vs 70). However, orchidectomies for testicular cancers were reduced by 63% (3/8).

On further analysis, the 13 scrotal explorations performed in 2019 were all paediatric patients and were evenly spread across the March to May timeline. In 2020, 6 scrotal explorations were performed in early March prior to COVID-19 lockdown regulations, with only 3 scrotal explorations in the period after lockdown regulations were instated. All patients undergoing scrotal exploration were children.

Ureteric stone surgeries were similar between both timelines, with all surgeries evenly spread over the March to May period of both years.

## Discussion

The COVID-19 pandemic has brought with it significant challenges for health services worldwide. Fears of surges of patients leading to overwhelmed hospitals and critical care facilities mandated a reduction in elective and non-urgent activity. However, emergency and critical care cannot be deferred in this manner.

As has been reported in the literature, public fear of contracting the virus lead to a significant reduction in presentations to Emergency Departments [3]. Whilst some of this can be attributed to fewer non-emergent patients presenting, there is concern that fear of COVID-19 may have led to people with critical or time-sensitive illness to avoid presenting, or to have delayed presentation leading to actual or potential harm.

This has been demonstrated in the case of acute myocardial infarction, with fewer overall presentations, longer time from first symptom to presentation (potentially leading patients to miss the thrombolysis window), more complicated in-hospital stay and worse overall outcomes reported in the COVID era than in the corresponding period in 2019 [4].

Fewer presentations have also been reported in the field of acute stroke [5–7]. Whilst overall numbers were reduced, the numbers of patients with Large-Vessel Occlusion strokes remained the same [6], implying the reduction in numbers arose from a decrease in those with mild symptoms seeking care. Similar to the case of myocardial infarction, longer time from onset to presentation was seen, along with a decrease in the numbers of patients undergoing reperfusion therapy [7].

The effect of the pandemic on acute urologic care has been reported by Porreca et al. [8] who showed reductions in presentation of all acute urological conditions, including those requiring urgent surgery, from the period before COVID to during the height of the pandemic. Motterle et al. interestingly observed significant reductions in overall consultations and emergency procedures performed, but noted an increased admission rate of those presenting acutely [9]. Similar findings were reported by Madanelo et al. in Portugal [10]. This may be attributed to patients who deferred seeking care being more unwell when they do present, or may reflect the fact that those with severe illness will continue to present, whilst those with more minor complaints will not.

We chose to assess the emergency urological surgical workload in our unit, over a 3-month period in the COVID era and compared it to the same 3 months in 2019. We specifically focused on Ureteroscopy and Scrotal Exploration as the procedures of interest.

All patients presenting acutely with ureteric colic and scrotal pain were deemed to be urologic emergencies, and the urgency with which they were treated was not effected by overall reductions in service due to COVID-19. This is in line with guidance published by Stensland et al., Cleveland Clinic and Guys and St. Thomas Hospital, who all classify these as Emergency procedures, which cannot be cancelled in an escalate-down scenario [11–13].

Our hypothesis was that owing to the severity of pain seen in ureteric colic, patients would continue to present to hospital, regardless of the pandemic, and that this would serve as a control group. With regard to scrotal exploration, owing to the paediatric population seen in our unit, we have low threshold for surgical exploration of scrotal pain, with most explorations being negative for testicular torsion. As such, we postulated that patients with less severe pain symptoms would present less frequently to the ED, or to General Practitioners who refer onward.

Our data aligned with our hypothesis; in that, the number of patients requiring surgical intervention for the management of ureteric calculi remained the same, whilst those undergoing exploration for acute testis pain were lower than the equivalent period in 2019.

What remains unclear, however, is the number of patients with acute testis pain who did not present despite suffering from a genuine testicular torsion. It is conceivable that there are patients in the community with as yet undiagnosed ischaemic and non-viable testes as a result of reluctance to present to hospital or their GP.

## Conclusion

The COVID-19 pandemic has brought with it significant changes in the patterns of presentation of acute urologic emergencies. Whilst patients with ureteric colic continue to present, those with acute testis pain requiring exploration attend less frequently, raising the possibility of undiagnosed testicular torsion in the community.
